# A retrospective study of inpatients diagnosed with degloving skin and soft tissue injuries

**DOI:** 10.1038/s41598-024-52171-8

**Published:** 2024-01-29

**Authors:** Shao-shuo Yu, Zhe Zhu, He Fang, Yao-nan Jiang, Chen-qi Tang, Ying Shi, Lan-xia Gan, Hong-tai Tang, Hai-bo Wang, Yu Sun, Zhao-fan Xia

**Affiliations:** 1 Department of Burn Surgery, The First Affiliated Hospital of Naval Medical University, Burn Institute of PLA, Research Unit of Key Techniques for Treatment of Burns and Combined Burns and Trauma Injury, Chinese Academy of Medical Sciences, Shanghai, People’s Republic of China; 2 Department of Hepatobiliary Surgery, PLA Naval Medical Center, Shanghai, China; 3 The 92493 Hospital of the Chinese People’s Liberation Army, Huludao, People’s Republic of China; 4 China Standard Medical Information Research Centre, Shenzhen, People’s Republic of China; 5 Clinical Trial Unit, First Affiliated Hospital of Sun Yat-Sen University, Guangzhou, People’s Republic of China; 6 Centre for Data Science in Health and Medicine, Peking University, Beijing, People’s Republic of China

**Keywords:** Trauma, Epidemiology

## Abstract

The overall picture of degloving skin and soft tissue injuries (DSTI) remains a blank space in China. Therefore, a retrospective study was designed to summarize the current situation of this injury. Patients diagnosed with DSTI hospitalized between 2013 and 2018 were identified from the Hospital Quality Monitoring System (HQMS) database, of whom demographics, injury characteristics, hospitalization and cost information were analyzed. A total of 62,709 patients were enrolled in this study. Male sex predominated, with a mean age of 43.01 ± 19.70 years. Peasants seemed to be the most vulnerable. East China and Hubei province had the most patients. The most and least frequently injured anatomic site were lower extremity and torso, respectively. Traffic-related accidents and summer accounted for the highest proportion in terms of injury mechanism and season. The operation rate of DSTI roughly showed a growing trend, and the average length of stay was 22.02 ± 29.73 days. At discharge, 0.93% of DSTI patients ended up in death. Medicine accounted mostly for hospitalization cost, while the proportion decreased year by year. More than half DSTI patients paid at their own charge. This study made a relatively detailed description of DSTI patients nationwide, and might provide enlightenments for better prevention and treatment.

## Introduction

Degloving skin and soft tissue injury (DSTI) is a kind of serious lesion in the field of surgery. The injured skin and the underlying tissue can peel off from the subcutaneous fascia or muscle layer under a sudden strong shear force^1,2^, sometimes accompanied by extensive damage to inside structures such as muscle, blood vessels, nerves and bones^3^. Patients with severe DSTI may suffer from complications like shock or infection, which can prolong the length of treatment and pose risk of disability or death^4^, rendering great losses^5^. Therefore, it is very important to find efficacious therapies for DSTI in traumatology and other pertinent subjects. As nationwide investigation on DSTI is sparse^6^, there is a lack of comprehensive understanding of the overall characteristics of this injury. Most researches focused on a particular body part aiming to highlight the importance of immediate and better treatments^7–10^. Here, based on data of national level, we designed this study to retrospectively analyse the demographics, injury characteristics, hospitalization and cost information of inpatients with DSTI in China from 2013 to 2018, hoping to provide a whole picture and some enlightenments for better prevention and treatment.

## Materials and methods

The current report is a retrospective study of the recent statistics related to degloving skin and soft tissue injuries, containing demographics, injury characteristics, hospitalization and cost information.

### Data sources

The Hospital Quality Monitoring System (HQMS) database is a mandatory patient-level national database system for inpatients authorized by National Health Commission. All tertiary hospitals in China are requested to submit standard electronic inpatient discharge records on a daily basis to HQMS. The front page of each hospital medical record is filed by doctors in charge. Then it is coded by professional coders based on the International Classification of Disease-10 (ICD-10).

For each inpatient, HQMS provide demographic information, clinical diagnosis, cause of injury or poisoning, treatment procedures, duration of hospitalization, cost information, etc. This study was approved by Shanghai Changhai Hospital Ethics Committee. The whole process of data acquisition and analysis complied with relevant regulations and guidelines. Informed consent was obtained from all subjects and/or their legal guardian(s).

### Inclusion and exclusion criteria

In this study, inpatients’ information on the front page recorded by HQMS was searched from January 1, 2013 to December 31, 2018. The inclusion criteria were defined as inpatients whose main discharge diagnosis on the front page of their medical records were degloving injuries, which were coded by anatomic site in the S and T segment of ICD-10 codes, including degloving injury of scalp, waist and back, the upper extremity, hand, the lower extremity, thigh, calf and foot. A total of 16 diagnostic codes were included in ICD-10 codes. All related ICD codes in this report were listed in Supplementary Table 1. When retrieving the distribution of patients for a particular classification, some data were shown “missing”, these would be excluded to ensure comparability.

### Indicators

Demographics (gender, age, occupation, district, province), injury characteristics (admission way and season, injury site, injury mechanism, comorbidities), hospital treatment (operation rate, operation type, operation frequency, length of stay, complications, nosocomial death) and cost information (overall expense, cost type, mode of payment) were collected for analysis, of which some hospital treatment-related indicators (the rate of operation, infection, shock, transfusion, ICU occupancy, nosocomial death, as well as LOS and hospitalization expense) were compared among different injury sites.

### Statistical analysis

Data were presented as means with SD for continuous variables and as frequencies with percentages for categorical variables. P values were not used because such a large sample size in HQMS would yield statistically significant p values with small absolute differences which may not be clinically meaningful. All statistical analyses were done using SAS V.9.4 (SAS Institute, Inc., Cary, North Carolina, https://www.sas.com/zh_cn/home.html)^11^.

### Ethical statements

According to ethical policies of Shanghai Changhai Hospital Ethics Committee, clinical data can be analysed and used under the precondition of without revealing the identity of patients.

## Results

### Demographics

**Figure 1:**
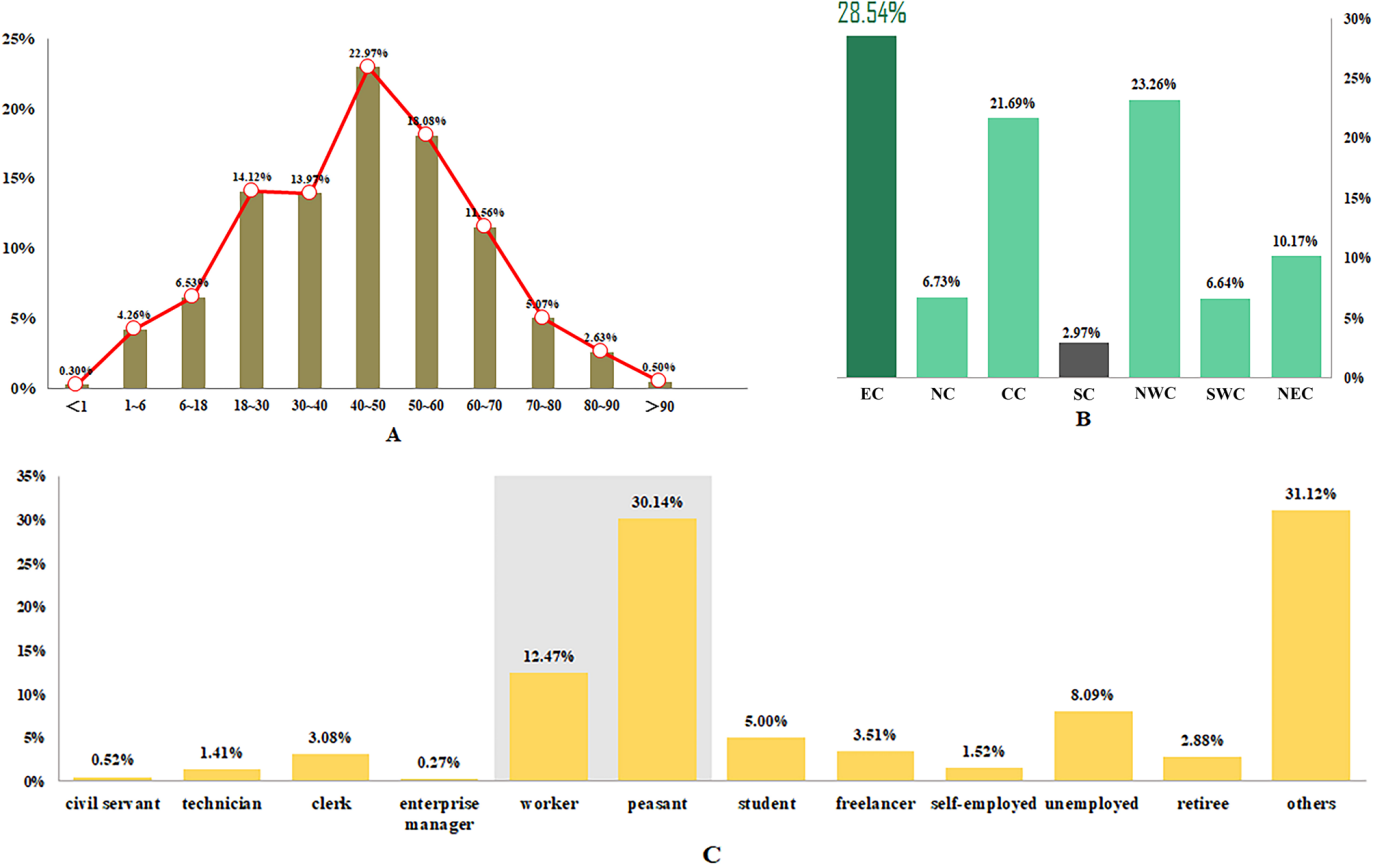
Demographics of DSTI inpatients from 2013 to 2018. (**A**) age, (**B**) district, (**C**) occupation. *EC* East China, *NC* North China, *CC* central China, *SC* South China, *NWC* Northwest China, *SWC* Southwest China, *NEC* Northeast China.

There were 62,709 patients diagnosed with DTSI, of whom 67.41% were males with a mean age of 43.01 ± 19.70 (Fig. 1A). Among them, East China ranked the first (28.54%) geographically (Fig. 1B), and Hubei province was at the top (11.45%) of all included provincial level administrative regions (Supplementary Fig. S1). The majority of DTSI patients were peasants (30.14%), followed by workers (12.47%) (Fig. 1C).

### Injury characteristics

**Figure 2:**
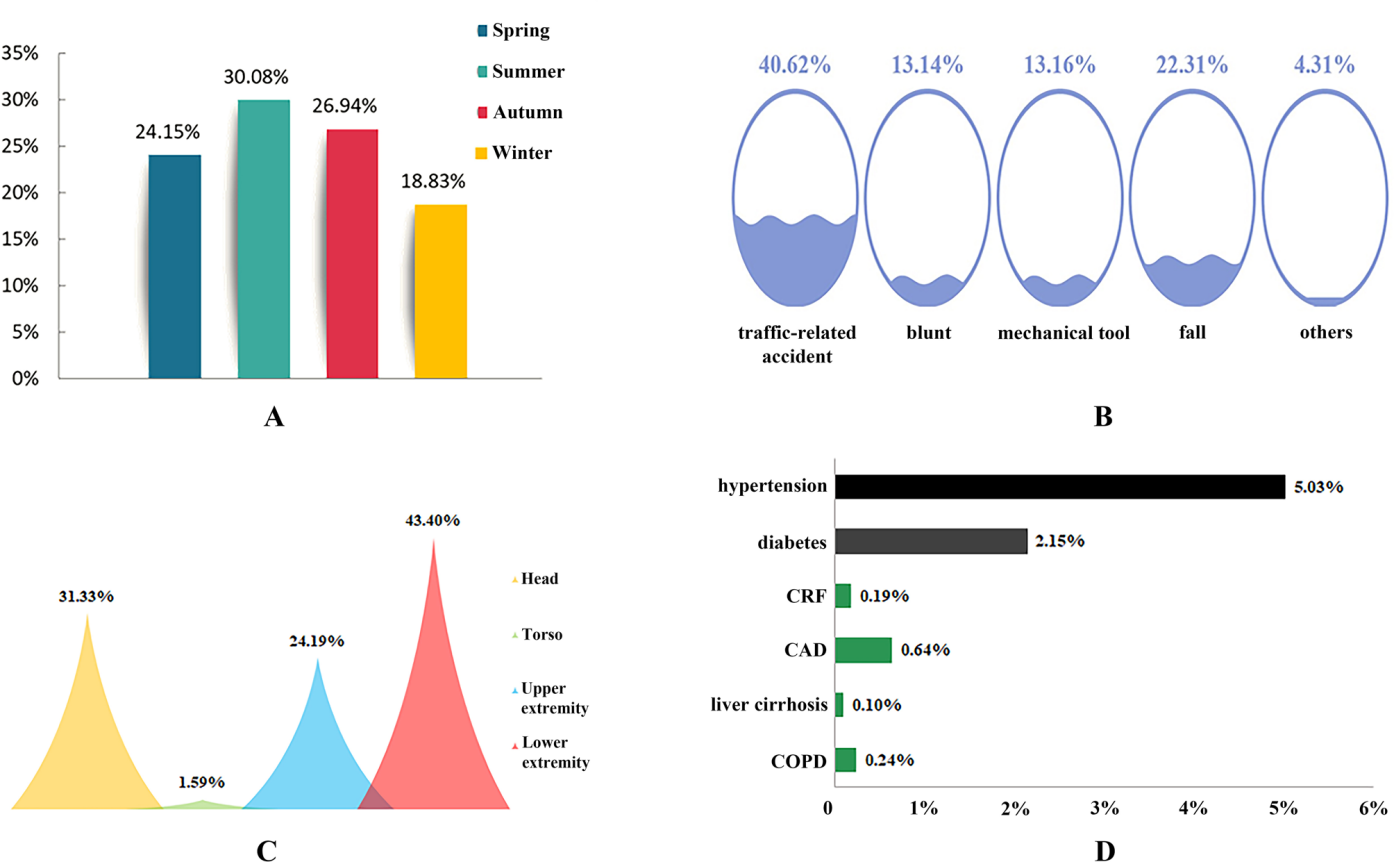
Characteristics of injury for DSTI inpatients from 2013 to 2018. (**A**) Season, (**B**) mechanism, (**C**) anatomical site, (**D**) comorbidity. *COPD* chronic obstructive pulmonary disease, *CAD* coronary artery disease, *CRF* chronic renal failure.

Patients diagnosed with DSTI were admitted to hospitals mainly through the emergency department (58.79%). Numbers of admissions peaked in summer (30.08%), followed by autumn (26.94%) and spring (24.15%) (Fig. 2A), with the majority concentrated in July (10.28%) and August (10.63%) (Supplementary Fig. S2). Traffic-related accidents were the leading mechanism of injury (40.62%), followed by falls (22.31%) (Fig. 2B). According to the diagnostic ICD-10 codes, DSTI of different anatomic sites were classified into eight categories, namely, degloving (skin/ soft tissue) injury of scalp, waist and back, the upper extremity, hand, the lower extremity, thigh, calf, foot. In order to be more scientific and reasonable, we combined these sites into four groups, that was, head (DSTI of the scalp), torso (DSTI of waist and back), the upper extremity (including DSTI of the upper extremity and hand) and the lower extremity (including DSTI of the lower extremity, thigh, calf and foot). The most frequently injured anatomic site was the lower extremity (43.40%), followed by head (31.33%), the upper extremity (24.19%) and torso (1.59%) (Fig. 2C). Comorbidities were found in 8.34% of all the inpatients, with hypertension being the most common (5.03%), followed by diabetes (2.15%) (Fig. 2D).

### Hospitalization

**Figure 3:**
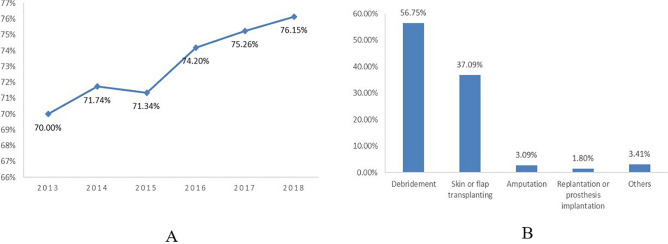
Operation rate and way of DSTI inpatients from 2013 to 2018. (**A**) Operation rate, (**B**) Operation way.

**Figure 4:**
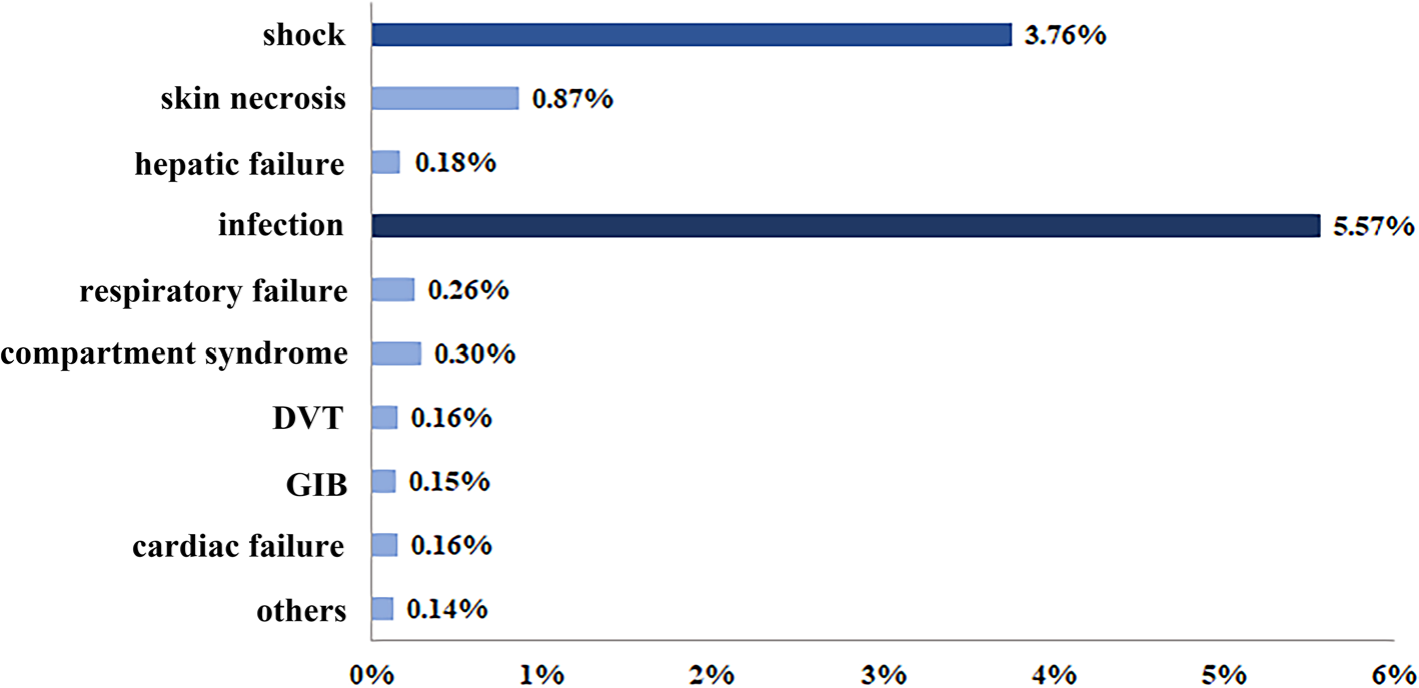
Incidence rate of complications of DSTI inpatients from 2013 to 2018. *DVT* deep venous thrombosis, *GIB* gastrointestinal bleeding.

The operation rate of DSTI roughly showed a growing trend year by year (Fig. 3A), with a mean of 73.30%, of which 37.09% were related to skin or flap transplanting (Fig. 3B) and 31.93% underwent multi-operations. The average length of stay was 22.02 ± 29.73 days. During hospitalization, 0.93% of DSTI patients ended up in death, and 12.13% developed complications, including infection (5.57%) and shock (3.76%), which happened far more often than the others (Fig. 4).

### Cost information

**Figure 5:**
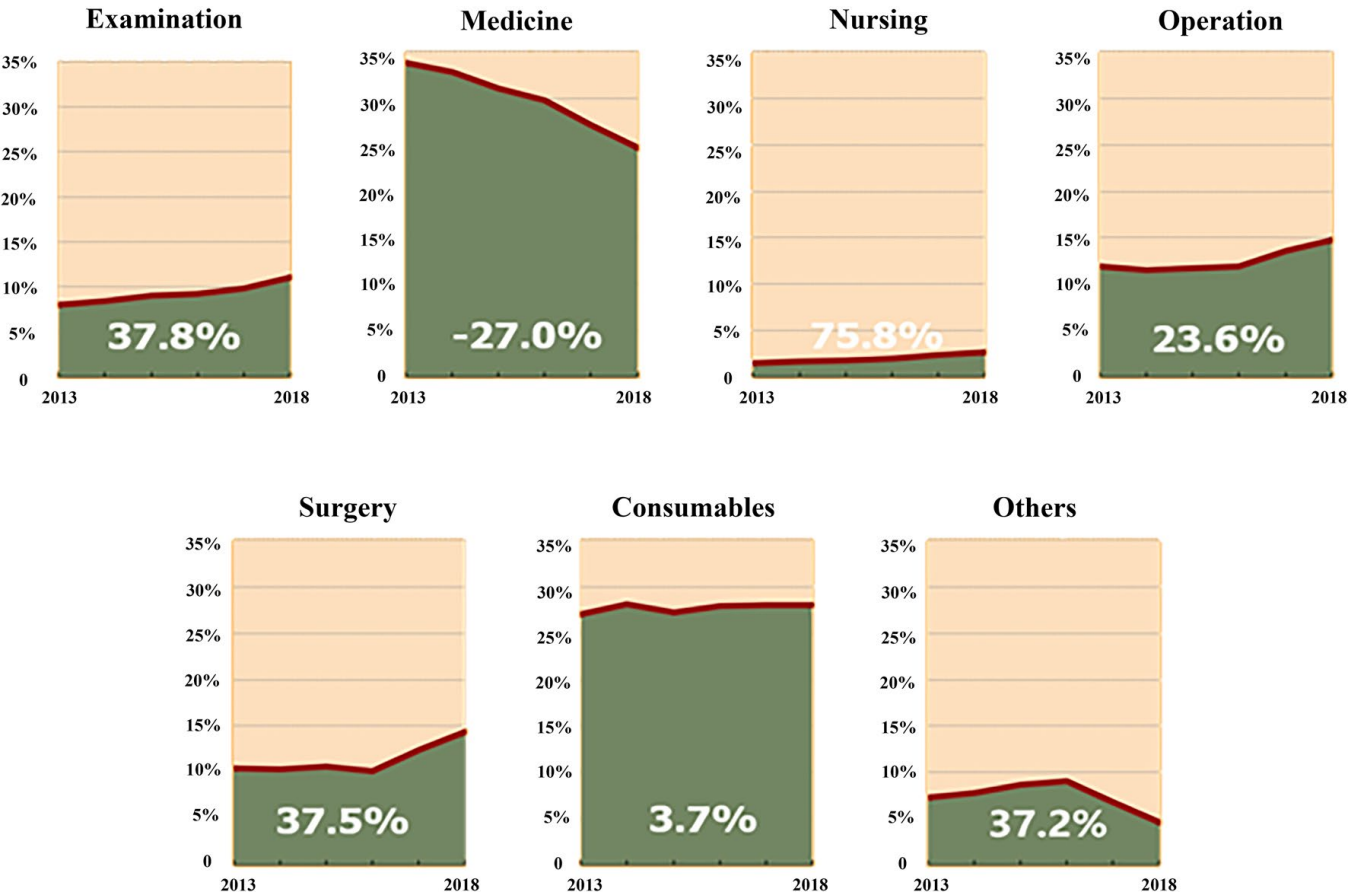
Hospitalization cost distribution of DSTI inpatients from 2013 to 2018.

**Figure 6:**
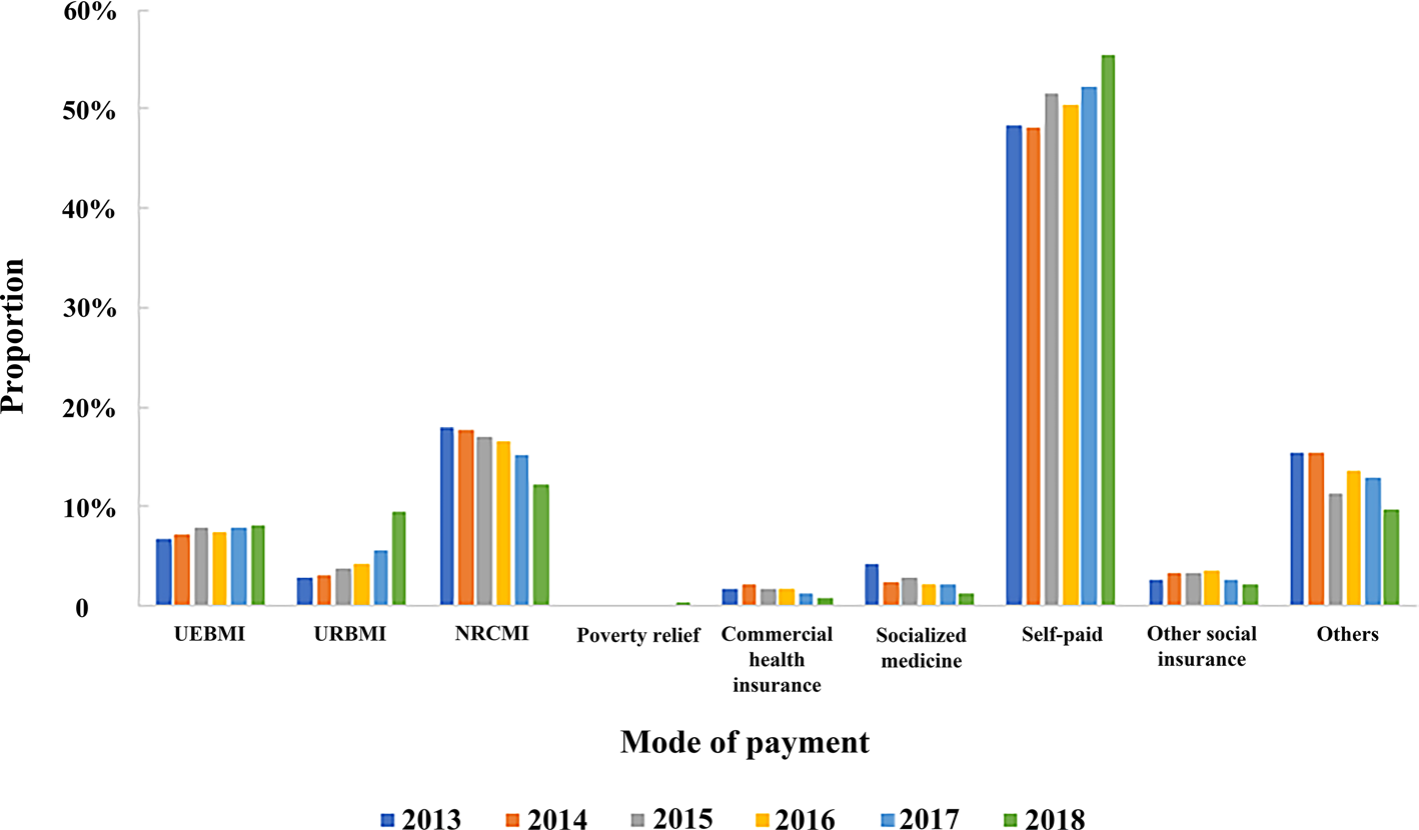
Payment mode of DSTI inpatients from 2013 to 2018. *UEBMI* Urban Employees’ Basic Medical Insurance, *URBMI* Urban Residents’ Basic Medical Insurance, *NRCMI* New Rural Cooperative Medical Insurance.

The average hospitalization expense of DSTI patients was 33.51 ± 53.59 thousand Chinese yuan. Medicine and consumables accounted for the largest proportion (59.57%), but the former was decreasing markedly year by year, while growing trends emerged to varying degrees among the rates of surgery, operation, nursing and examination expenses, respectively (Fig. 5). More than half of the patients paid at their own charge (51.10%), which was rising together with that from urban medical insurance. Those supported by the New Rural Cooperative Medical System had gradually decreased in recent years (Fig. 6).

### Characteristics of different injury sites

Hospitalization treatment-related variables were then compared among the four different injury sites. Patients with the upper extremity DSTI seemed to have the best outcomes, who were more likely to undergo surgery, but had lower transfusion rate and ICU occupancy, as well as low incidence of complications like infection or shock, with significantly shorter LOS, less expenditure and lower nosocomial mortality. DSTI in the lower extremity needed more operations, with high transfusion rate, long hospital stay and correspondingly high cost. Though the incidence of DSTI in the torso was low, it showed high rate of shock and infection. Patients with head DSTI had the lowest operation rate, with the highest ICU occupancy and hospital mortality (Table 1).

**Table 1:** Data of DSTI patients with different anatomical sites affected.

	Head	Torso	Upper extremity	Lower extremity
Number	19,647	997	15,168	27,214
LOS (day)	18.33 ± 23.65	23.36 ± 30.20	16.84 ± 20.51	27.84 ± 36.46
Infection (%)	6.72	7.02	2.44	6.48
Shock (%)	4.51	5.32	1.46	4.54
Operation (%)	61.94	66.5	80.96	77.47
Operation frequency (proportion %)				
1	78.13	66.67	75.69	57.87
> 1	21.87	33.33	24.31	42.13
Transfusion (%)	21.6	21.97	10.36	24.98
ICU occupancy (%)*	7.07	6.02	1.42	4.05
Nosocomial death (%)	1.57	0.90	0.33	0.83
Hospitalization cost (CNY)	27,068.07 ± 41,919.14	39,856.09 ± 77,807.4	23,913.53 ± 37,458.69	43,572.07 ± 64,364.89

*ICU occupancy rate: number of DSTI patients who were treated in Intensive Care Unit/the total number of DSTI patients × 100%.

## Discussions

As the largest organ covering the surface of human body, our skin is the first protective barrier to resist external undesirable stimuli. It also has multiple functions such as excretion, temperature regulation and sensation, making it an important structure to maintain the internal stability of the organism. The prevalence of DSTI is difficult to learn for sure, as large-scale multi-center studies are rare. In this report, the incidence was 0.58%. In a similar study focused on trauma patients in 2019, no description of degloving injuries was found. But it did show top ten trauma-related diagnosis of all trauma patients, and DSTI was not among them^12^. Therefore, it can be estimated that the incidence of DSTI was not high compared with other traumatic injuries. An investigation pertaining to skin injury prevalence and incidence in China enrolled 13,176 patients across nine tertiary hospitals, and showed a prevalence rate of 1.07% in skin tears^13^, much lower than that in other countries, which rate was between 3.9 and 22%^5,12,14,15^. This may result from differences in the study populations, as those study samples were mainly from the elders in health care settings. As an acute injury, DSTI would most likely to be witnessed first at the emergency department. Our result confirmed this, as more patients were admitted through the emergency department. Due to the varying severity of DSTI, some patients with mild symptoms might go home directly after emergency treatment and did not need hospitalization. Therefore, a potentially large number of patients in the emergency medical record system, whose information cannot be retrieved through HQMS, may have been omitted, leading to the underreported incidence.

In spite of the low incidence, the rising trend of aging and trauma-caused disability-adjusted life years and the resultant social and family burdens make DSTI impossible to be underestimated^16^. Latifi et al. proposed that the severity of DSTI mainly depends on the mechanism of injury, comorbidities (especially diabetes), concomitant injuries, injury site and type (open or closed)^4^. Early diagnosis of DSTI is essential, as inadequate or delayed treatment would lead to infection, turning the injury to chronic wounds, especially for those with poor health status^17^. Health care resources such as wound dressings would be consumed by a large number^18^, and duration of hospitalization wound increase^5^. But DSTI can occur on any parts of body and in any population^17^, and there is no established guidelines or consensus on an appropriate treatment, therefore, prevent and efficacious management is challenging^19^. For many years, epidemiological investigation and detailed analysis have been carried out in this field^4–6,14,15,19,20^, yet there is a paucity of similar large-scale research in China. The 2019 multicenter study included only 141 skin tear cases across nine hospitals, so the sample size was too small to be nationally representative^21^. This study is the first retrospective one that targeted a nationwide, multicentric data bank to make a relatively detailed description of demographic and medical information of degloving skin and soft tissue injuries during recent years in China. Our study shows that DSTI is more common in middle-aged males, and the leading mechanism of injury is traffic-related accidents, which is consistent with previous conclusions from similar studies in other countries^19,22–24^, but inconsistent with those involving skin tears^14,15^. This disparity stems mainly from definition difference. Skin tear, as its generally accepted definition by Payne and Martin, is “a traumatic injury occurring principally on the extremities of older adults as a result of shearing or friction forces which separate the epidermis from the dermis (partial-thickness wound) or which separate both the epidermis and the dermis from underlying structures (full-thickness wound)”^25^. As this study focused on a larger population with broader inclusion criteria, our results differed a lot from the formers. The lower incidence of DSTI in winter may be attributed to more clothes, as they decrease direct exposure of the skin^26,27^. Specific skin diseases, which change the status of skin, have seasonality. Xerosis, which is more prominent in the colder months, may act as a potential mediating factor contributing to skin tears^21^. The number of rural patients (NRCBI) has been decreasing, while urban patients and those who can pay medical expenses at their own charge keep rising, which should be attributed to the acceleration of China's urbanization process and the improvement of people's economic level.

Current literature on DSTI is mostly based on specific anatomical sites. In this study, we found that the incidence of DSTI in lower extremities is the highest, somewhat in accordance with previous studies^4,5,17,19,28,29^. Notably, severe concomitant injuries could be the main lethal factors in some fatal cases^19^. For instance, the rate of ICU occupancy and nosocomial mortality of DSTI in the head were higher than the other sites despite of its secondary incidence, which is owing to many traumatic brain injuries occurred along with DSTI, eliciting increased deaths. Although DSTI in torso was the least common, it had the highest complication rate of infection and shock. This result can be interpreted in two aspects. On the one hand, there is a good chance that patients diagnosed with DSTI of the torso also suffered from chest and abdominal viscera injuries, which caused heavy bleeding and increase the risk of infection. On the other hand, compared with head and limbs, the circulation of skin in torso is less sufficient, combined with the limitation of patients’ position, which can make rehabilitation process harder. Of the four anatomical sites in our classification, DSTI of the upper extremity had the best treatment outcomes, with fewer complications and significantly shorter LOS, less expenditure and lower nosocomial mortality. Considering the indispensable functions of the upper extremity, particularly the hands, it is very reasonable to actively perform operations on them. Furthermore, the upper extremity is relatively small in area and rich in blood supply, plus the slightly less difficulty in postoperative nursing, so it has faster recovery and better prognosis. In a 10-year analysis of skin tear in an Australian acute care hospital, most STAR category 1A/1B and STAR category 2A/2B skin tears occurred on the upper extremities, while STAR category 3 wounds occurred mostly on the lower extremities^5^, indicating that injuries to the upper limbs were minor. Though there was no defined severity rating in our study, we speculated that DSTI of the upper extremity in our study might also fit with these characteristics. The continuity of great vessels and capillary beds can be disrupted when DSTI occurs, followed by the formation of edema, which increase the diffusion distance of oxygen. The injured tissue swells within a confined space and becomes ischemic, creating a vicious cycle that leads to serious complications such as compartment syndrome^30^. Necrosis of the skin can occur in the degloved area due to direct damage to the cutaneous layers or swelling of the subcutaneous tissue^31^. These are generally considered to be serious complications of DSTI. Once they occur, the complexity and difficulty of treatment will increase dramatically. In this study, the incidence of skin necrosis and compartment syndrome was 0.87% and 0.30%, respectively. Less than the proportion of infection and shock, though, it surpassed the concurrent rate of other complications, which should be noticed by surgeons. In addition to the routine prevention and treatment of common complications when managing DSTI, these two kinds of complications should be cautious about in case they occur and impair skin condition.

As this is the first time to carry out the big data analysis of DSTI, problems are inevitable. It is known that the trauma severity score (ISS/AIS)^19,31,32^, which is widely used internationally to evaluate the severity of trauma on patients’ first admission, has been a vital reference for guiding later treatment and predicting the prognosis. And the STAR Classification System^33^, a validated skin tear classification system, has a targeted significance for assessing the severity of DSTI. However, lack of such evaluation indexes in HQMS makes it impossible for analysts to directly assess the severity of injury, instead we can only make predictions from indirect materials (length of stay, transfusion rate, ICU occupancy rate, complication rate, etc.), which affects the accuracy of final conclusions. If ISS/AIS could be added to the upgraded system subsequently or the severity could be directly recorded on the front page by physicians after DSTI was diagnosed, it would play a positive role in promoting more scientific and effective diagnosis and treatment of DSTI, even for all the trauma patients.

Restricted by a variety of subjective and objective factors, there are inevitably some deviations in data screening, sorting and analysis in this study. On the premise of not distorting the final results, we’ve discarded the data that was incomplete or obviously divergent from the reality, and made recommendations to departments concerned on the current situation of non-standard information collection on the front page of medical records. Patients of different ages have their own characteristics in the clinical manifestations and prognosis of diseases due to the inherent disparities. By stratifying them according to their ages and making contrasts, we can expect a promising prospect when diagnosis and treatment of patients with trauma can be carried out in a more scientific way. More focus will be put on this in our future researches. As an injury of skin-related surgery, treatment of DSTI cannot be limited to a single subject^34^. Irreversible damage to the skin caused by deep wounds means later troublesome problems such as the formation of scar, which hasn’t yet been effectively resolved globally, and thus has long-term follow-up significance. This study is our first tentative exploration on the overview of DSTI in recent years in China. More in-depth investigation and discussion still need to be made in order to obtain more comprehensive understanding, and to provide more instructive conclusions for the prevention, diagnosis and treatment of DSTI as well.

## Conclusions

This is the first retrospective study that targeted a nationwide database to make a relatively detailed epidemiological analysis of degloving skin and soft tissue injuries from 2013 to 2018 in China. Through this work, we have not only obtained a preliminary understanding of DSTI and enlightenments for better prevention and treatment, but also guidance to the future in-depth studies in this field.

### Supplementary Information

Supplementary Figure S1.

Supplementary Figure S2.

Supplementary Table 1.

## Data Availability

The datasets generated during and/or analysed during the current study are available from the corresponding author on reasonable request.

## Abbreviations

DSTI: Degloving skin and soft tissue injury
HQMS: Hospital quality monitoring system
ICD: International classification of diseases
LOS: Length of stay
ICU: Intensive care unit
EC: East China
NC: North China
CC: Central China
SC: South China
NWC: Northwest China
SWC: Southwest China
NEC: Northeast China
COPD: Chronic obstructive pulmonary disease
CAD: Coronary artery disease
CRF: Chronic renal failure
DVT: Deep venous thrombosis
GIB: Gastrointestinal bleeding
UEBMI: Urban employees’ basic medical insurance
URBMI: Urban residents’ basic medical insurance
NRCMI: New rural cooperative medical insurance
ISS/AIS: Injury severity score/abbreviated injury score
