# Supplementary figures and images for: A retrospective study of inpatients diagnosed with degloving skin and soft tissue injuries
Source: Sci Rep. 2024 Jan 29;14:2392. doi: 10.1038/s41598-024-52171-8 (PMC10825152; doi:10.1038/s41598-024-52171-8)

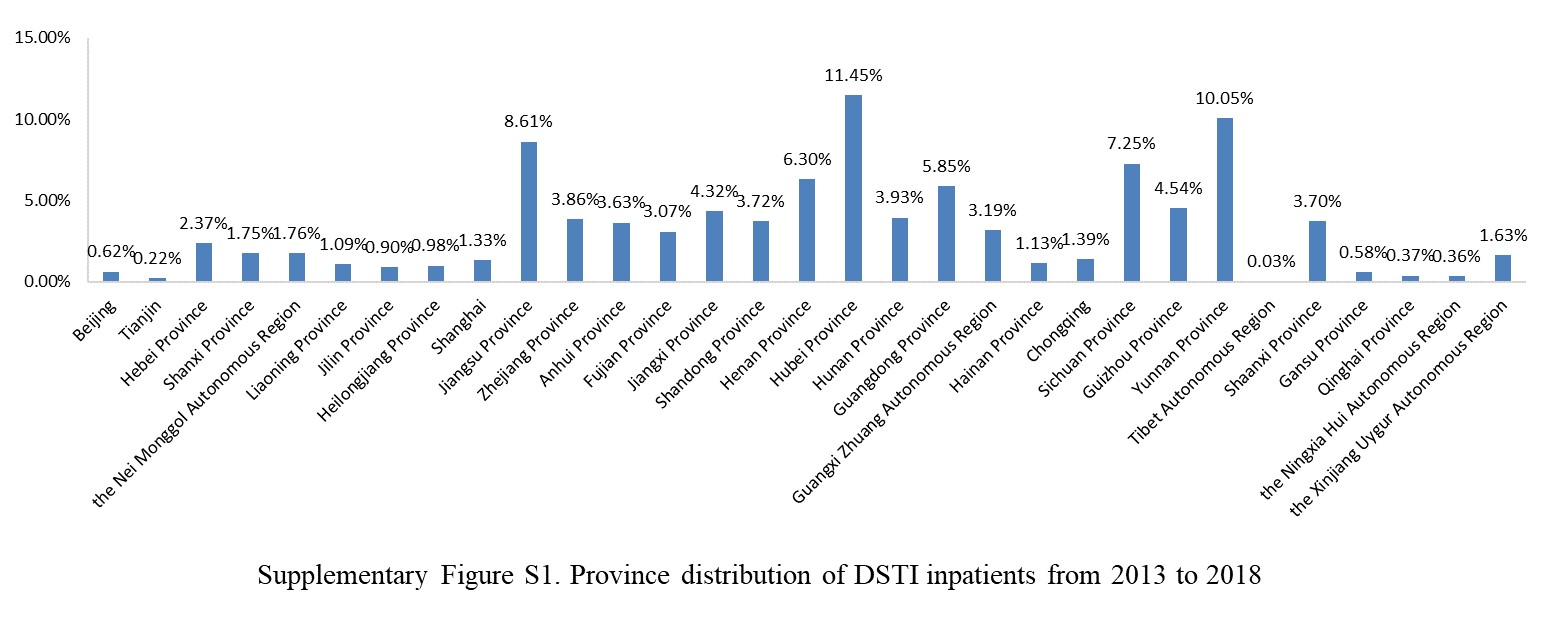

Supplement: Supplementary file 1 — Supplementary Figure S1. [file 41598_2024_52171_MOESM1_ESM.jpg]

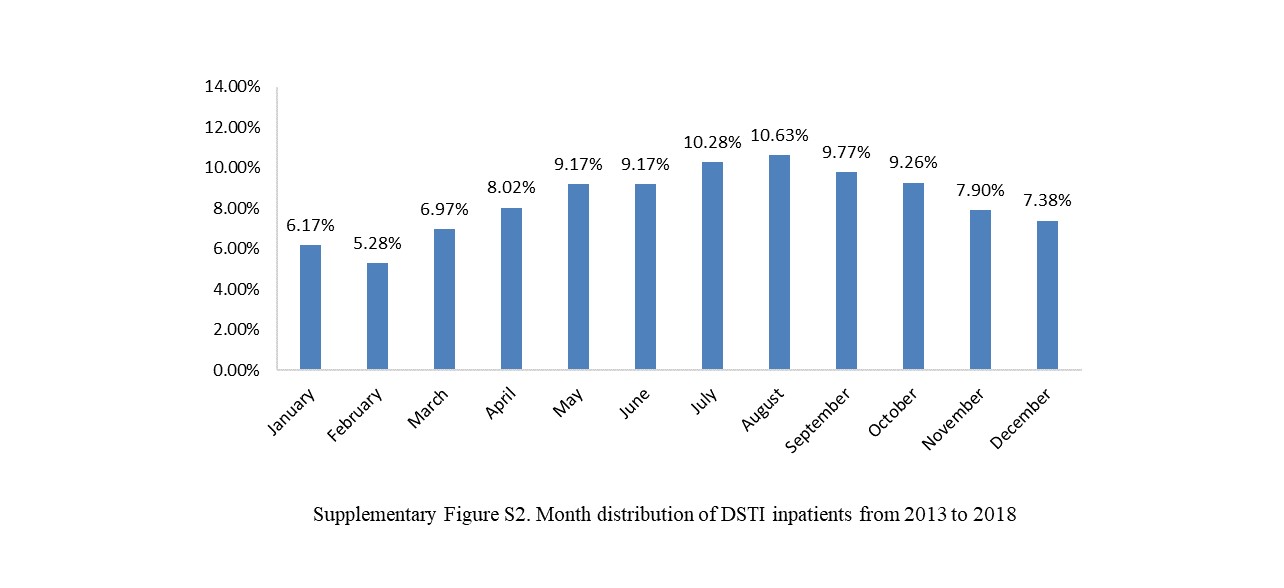

Supplement: Supplementary file 2 — Supplementary Figure S2. [file 41598_2024_52171_MOESM2_ESM.jpg]
