# Supplementary material for: A retrospective study of inpatients diagnosed with degloving skin and soft tissue injuries
Source: Sci Rep. 2024 Jan 29;14:2392. doi: 10.1038/s41598-024-52171-8 (PMC10825152; doi:10.1038/s41598-024-52171-8)
Supplement: Supplementary file 3 — Supplementary Table 1. [file 41598_2024_52171_MOESM3_ESM.pdf]

A Retrospective Study of Inpatients Diagnosed with Degloving Skin and Soft tissue Injuries

Shao-shuo Yu[1#], He Fang[1#], Yao-nan Jiang[1,2#], Zhe Zhu[1,3#], Chen-qi Tang[1], Ying Shi[4], Lan-xia Gan[4], Hong-tai Tang[1], Hai-bo Wang[5*], Yu Sun[1*] and Zhao-fan Xia[1*]

[1] Department of Burn Surgery, the First Affiliated Hospital of Naval Medical University, Burn Institute of PLA, Research Unit of key techniques for treatment of burns and combined burns and trauma injury, Chinese Academy of Medical Sciences, Shanghai, P.R. China

[2] The 92493 Hospital of the Chinese People's Liberation Army, Huludao, P.R. China,

[3] Clinic of the 91681 troop of the Chinese People's Liberation Army, Ningbo, China

[4] China Standard Medical Information Research Centre, Shenzhen, P.R. China

[5] Clinical Trial Unit, First Affiliated Hospital of Sun Yat-Sen University, Guangzhou, P.R. China; Peking University, Centre for Data Science in Health and Medicine, Beijing, P.R. China

[#] These authors contributed equally to this work and are considered to be co-first authors.

* Corresponding authors:

Zhao-fan Xia, Tel: (+86)2131161821, E-mail: xiazhaofan@163.com

Yu Sun, Tel: (+86) 13816873483, E-mail: littlefish0916@126.com

Hai-bo Wang, Tel: (+86)2131161821, E-mail: haibo@mail.harvard.edu

Supplementary Table S1. ICD-10 Code for Degloving Injuries Diagnosis

**Diagnosis of Degloving Injuries**	**ICD-10 encoding**
degloving injury of scalp	S08.000<br>S08.001
degloving skin injury of waist and back	S31.002
degloving injury of the upper extremity	T11.102
degloving injury of hand	S61.902<br>S61.900x005
Degloving (skin) injury of the lower extremity	T01.301<br>T13.103<br>T13.100x003
degloving injury of thigh	S71.101
degloving injury of calf	S81.901<br>S81.902
degloving (soft tissue) injury of foot	S91.305<br>S91.306<br>S91.300x002<br>S99.800x001
